# Contrast-optimized composite image derived from multigradient echo cardiac magnetic resonance imaging improves reproducibility of myocardial contours and T2* measurement

**DOI:** 10.1007/s10334-015-0503-6

**Published:** 2015-11-03

**Authors:** Pandji Triadyaksa, Astri Handayani, Hildebrand Dijkstra, Kadek Y. E. Aryanto, Gert Jan Pelgrim, Xueqian Xie, Tineke P. Willems, Niek H. J. Prakken, Matthijs Oudkerk, Paul E. Sijens

**Affiliations:** Center for Medical Imaging-North East Netherlands, EB45, University of Groningen, University Medical Center Groningen, PO Box 30001, 9700 RB Groningen, The Netherlands; Department of Radiology, EB45, University of Groningen, University Medical Center Groningen, PO Box 30001, 9700 RB Groningen, The Netherlands; Department of Physics, Diponegoro University, Prof. Soedarto street, Semarang, 50275 Indonesia

**Keywords:** Magnetic resonance imaging, Myocardium, Iron overload, T2* quantification, Contrast-optimized composite image

## Abstract

**Objectives:**

Reproducibility of myocardial contour determination in cardiac magnetic resonance imaging is important, especially when determining T2* values per myocardial segment as a prognostic factor of heart failure or thalassemia. A method creating a composite image with contrasts optimized for drawing myocardial contours is introduced and compared with the standard method on a single image.

**Materials and methods:**

A total of 36 short-axis slices from bright-blood multigradient echo (MGE) T2* scans of 21 patients were acquired at eight echo times. Four observers drew free-hand myocardial contours on one manually selected T2* image (method 1) and on one image composed by blending three images acquired at TEs providing optimum contrast-to-noise ratio between the myocardium and its surrounding regions (method 2).

**Results:**

Myocardial contouring by method 2 met higher interobserver reproducibility than method 1 (*P* < 0.001) with smaller Coefficient of variance (CoV) of T2* values in the presence of myocardial iron accumulation (9.79 vs. 15.91 %) and in both global myocardial and mid-ventricular septum regions (12.29 vs. 16.88 and 5.76 vs. 8.16 %, respectively).

**Conclusion:**

The use of contrast-optimized composite images in MGE data analysis improves reproducibility of myocardial contour determination, leading to increased consistency in the calculated T2* values enhancing the diagnostic impact of this measure of iron overload.

## Introduction

Myocardial T2* imaging has been adopted in clinical studies for non-invasive longitudinal assessment of myocardial iron deposition [1, 2]. Inferring iron deposition from magnetic resonance T2-weighted imaging requires the fitting of signal intensities from a region of interest (ROI) propagated through a series of multi-gradient echo (MGE) acquisitions to a T2* equation.

In myocardial T2* assessment, a single short-axis image that corresponds to a single echo time (TE) of a MGE series is generally selected to draw left ventricular (LV) epicardial and endocardial contours. Commercial software packages available for T2* calculation also rely on single image selection. Examples are the use of the first or second TE of the MGE image series [3] or a TE showing good contrast between the left ventricle blood pool (LVBP) and the myocardium [4].

The clarity of LV epicardial and endocardial contours depends on the contrast difference between the myocardium and LVBP defining its inner border and between the myocardium with right ventricle blood pool (RVBP), epicardial fat, abdominal fat, stomach, and lung defining its outer border [5, 6]. Reflecting the differences in transverse magnetization, a short-axis image acquired at short TE tends to have little contrast between LV myocardium and LVBP or RVBP, while at long TE it tends to show poor contrast between LV myocardium and lung. Images with long TE are subject to signal loss at regions adjacent to the posterior vein of the LV [7]. A single short-axis image cannot, therefore, provide optimal contrast between LV myocardium and all of its surroundings. Inaccurate, poorly reproducible myocardium contours are expected to result in unreliable T2* determination and, therefore, unreliable iron content representation [8].

In clinical practice, the T2* technique as a measure of myocardial iron accumulation is applied on ROIs covering either mid-ventricular septum [1] or the entire left ventricular wall on three short axis slices [2]. T2* assessment limited to the mid-ventricular septum rather than the global contour is meant to avoid susceptibility artifacts arising from the anterior and posterior cardiac vessels veins and the lung [1]. However, Meloni et al. [3, 9] showed that blood oxygenation does not significantly affect the global heart T2* values. Moreover, in a large study global heart T2* identified four groups of patients having different stages of myocardial iron loading in which the mid-ventricular septum had T2* values not matching those of the global heart [2].

The standard method for T2* assessment is manually drawing the myocardial contour on one visually selected MGE image. It is likely that contour reproducibility is affected by the choice of which MGE image is used for the tracing. As an alternative, we introduce a contrast-optimized composite image derived from those short-axis MGE images providing maximum contrast-to-noise ratio between LV myocardium and its main surrounding areas (left ventricular blood pool, right ventricular blood pool, and lung) as a new standard image in T2* assessment. The interobserver reproducibility of myocardial contouring in the T2* assessment by this new method is tested by comparing the outcomes to those made by the established manual standard method for global ROI contours as well as those limited to the mid-ventricular septum. Therefore, the purpose of this study is to provide a method for improving reproducibility of myocardial contour and T2* determination in MGE bright blood magnetic resonance imaging.

## Materials and methods

### Study population

This study was approved by the hospital review board, which waived informed consent since the study was retrospective and involved postprocessing of clinical data. Between February 2009 and May 2011. Twenty-one consecutive patients were examined by MGE cardiac magnetic resonance imaging in a clinical routine, including nine haematology patients (4 male, mean age 36 ± 22 year) for whom determination of T2* as a measure of myocardial iron content was requested, and twelve patients suspected of cardiomyopathy (8 male, mean age 45 ± 18 year). No patients were excluded.

### Magnetic resonance

**Fig. 1:**
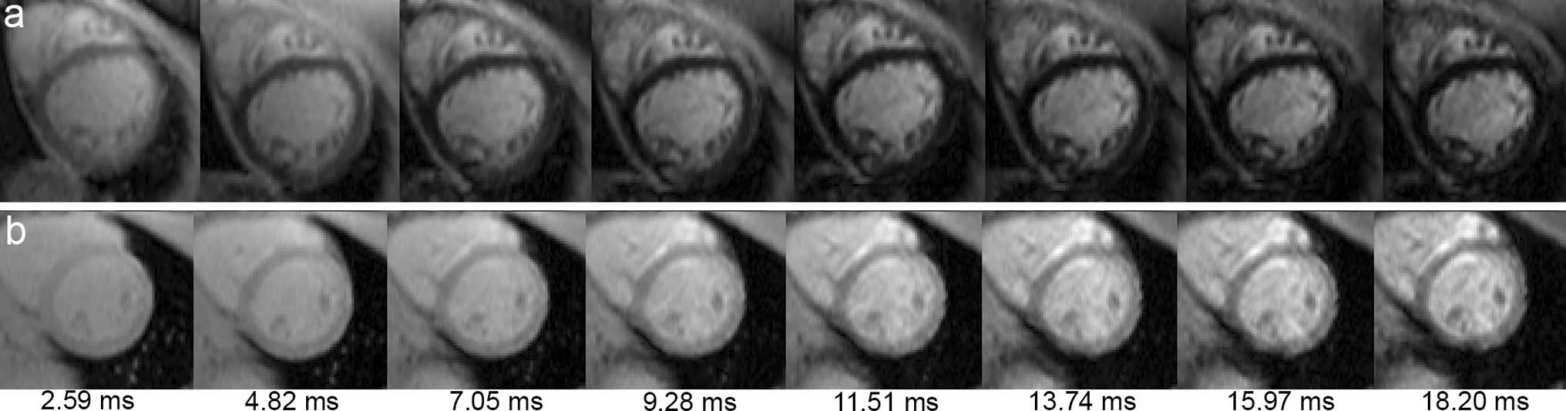
Multigradient echo series of two mid-ventricular short-axis myocardia with the lowest calculated segment T2* value of 8.20 ms represents iron loading (*series a*) and 24.27 ms indicates lack of iron (*series b*). Visual image selection (method 1; blind, independent selection by each observer) meant selection of the image where the LV myocardial wall was clearest; in *series a*, all four observers chose TE at 4.8 2 ms while in *series b*, two observers chose TE at 4.82 and 9.28 ms while the other two chose TE at 15.97 ms

Patients were examined by cardiac magnetic resonance at a 1.5 T whole body scanner (Siemens Avanto, Erlangen, Germany) using a standard body matrix radio frequency (RF) coil of six elements and a spine matrix coil of 6–24 elements depending on the chosen field of view. A bright blood MGE sequence with a single breath hold was performed at 8 TE (2.59–18.20, and 2.23 ms increment), repetition time 200 ms, flip angle 20°, variable field of view (237–400) × 400 mm^2^ depending on patient size, reconstructed voxel size 1.56 × 1.56 × 10 mm^3^, 50 % phase resolution sampling, phase encode per cycle 5, or 16–26 cardiac cycles per breath hold, pixel bandwidth 814 Hz, and without enabling parallel imaging. In the analyses described in "Reproducibility evaluation of myocardial contouring", raw image data were used. Electrocardiogram (ECG) triggering, breath-holding together with an acquisition window shorter than the diastole duration were set to prevent motion blurring. From nine haematology patients, a total of twenty-three LV short-axis slices were acquired at basal, mid-ventricular, and apical positions of seven patients and at two mid-ventricular slices of two patients. From twelve suspected cardiomyopathy patients, eleven short-axis at mid-ventricular from eleven patients and two at mid-ventricular and basal from one patient were acquired, adding up to 36 LV short-axis slices in total. A typical dataset for mid-ventricular short-axis slice is shown in Fig. 1.

### Composite image formation

A custom-written software for myocardial T2* assessment was developed in MATLAB version 7.14 (The MathWorks, Natick, MA, USA). The software has the option to assess the T2* by manually drawing epicardial and endocardial contours on one subjectively selected TE image of MGE image series (Fig. 1) and on a contrast-optimized composite image.

**Fig. 2:**
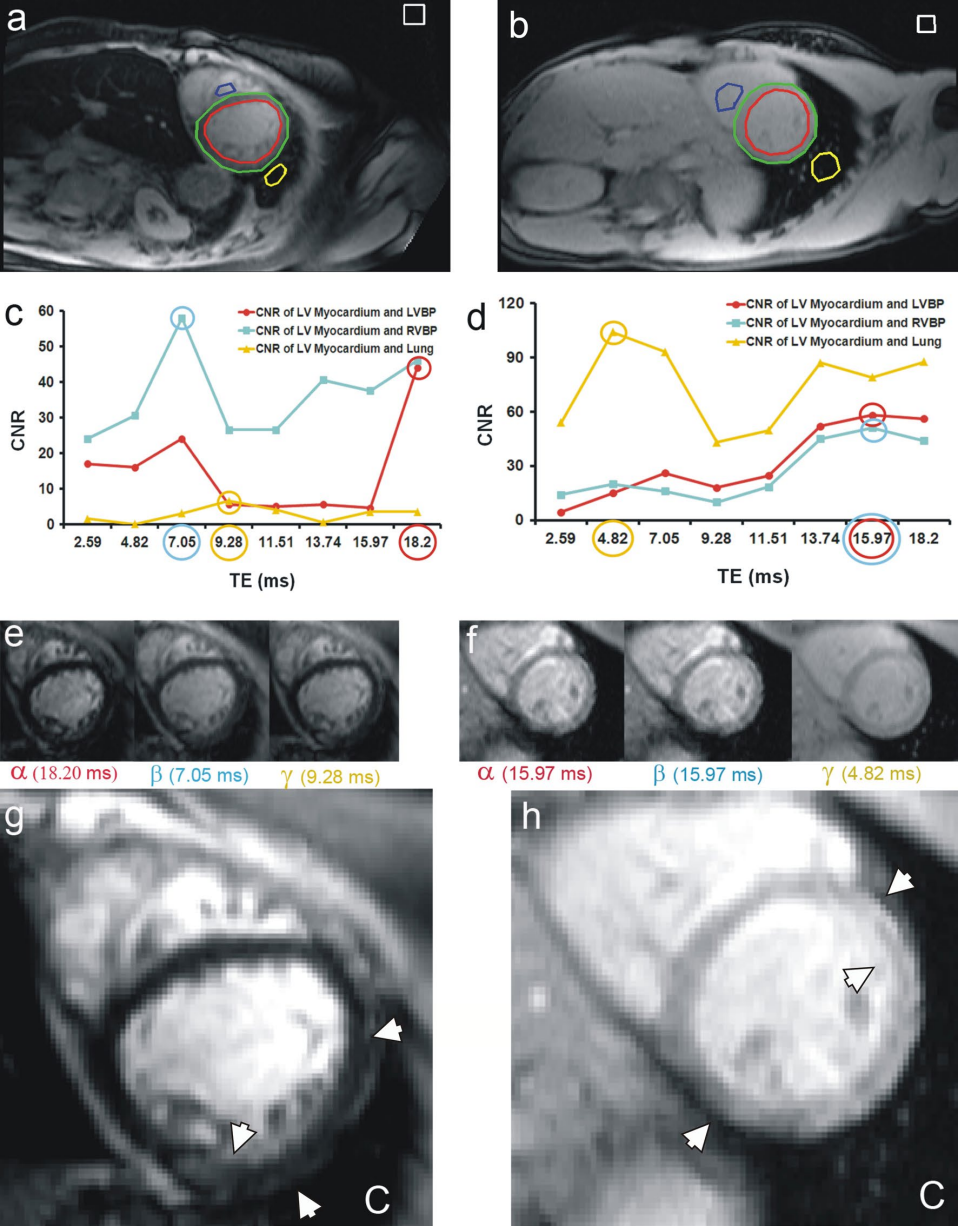
Composite image generation (method 2) as shown by two patients with (**a**, **c**, **e**, **g**), and without iron loading (**b**, **d**, **f**, **h**). First, the regions of interest (ROI) are drawn manually on the epicardial left ventricle myocardium (*green*), left ventricle blood pool (*red*), right ventricle blood pool (*blue*), lung (*yellow*), and air background (*white*) (**a**, **b**). Then the contrast-to-noise ratio (CNR) of the ROIs of the multi gradient echo (MGE) image series are calculated and plotted (*red*: LV myocardium-LVBP, *blue*: LV myocardium-RVBP, *yellow*: LV myocardium-lung) with optimal CNR values and the corresponding echo times are highlighted by *circles* (**c**, **d**). Three MGE images with optimal CNR, as a representation of α, β, γ, are then composed according to Eq. 3 (**e**, **f**). The composite image corresponding to C in Eq. 3 gains contrast and better demarcation, especially at the free wall of the myocardium and reduces the appearance of gradient echo susceptibility artefact at the inferior demarcation as highlighted by *arrow heads* (**g**, **h**)

The contrast-optimized composite images were generated first by manual drawing, on the first TE of the MGE image series, a contour of epicardial left ventricular myocardium, LVBP, RVBP, and lung with avoiding blood vessel, and on air background staying clear of any visible artefact (Fig. 2a, b). The ROI between the epicardial LV and LVBP was determined as the region of the myocardium. All contours were automatically propagated through the MGE image series and the signal-to-noise ratio (SNR) of myocardium was calculated using the following equation [10]:

1 $${\text{SNR}} = {\text{NF}}\frac{\text{SI}}{{\sigma_{b} }},$$

where NF, SI, *σ*_*b*_ represent the noise factor, signal intensities of the full myocardial contour representing the signal intensity of myocardium at every TE and standard deviation of air background, respectively. The noise factor in this equation accounts for the underestimation of noise derived from magnitude data and varies between 0.655 for a single RF coil and 0.71 for up to 32 coil elements [11]. Next, the contrast-to-noise ratios (CNRs) in each MGE image were calculated by using the following equation [10]:

2 $${\text{CNR}} = {\text{NF}}\frac{{|{\text{SI}}_{s} - {\text{SI}}_{m} |}}{{\sigma_{b} }} ,$$

where NF, SI_*s*_, SI_*m*_, and *σ*_*b*_ represent the noise factor, signal intensities of the surroundings boundary of LV myocardium (LVBP [inner], RVBP [septal], and lung [anterolateral]), LV myocardium, and standard deviation of air background, respectively.

Images with the highest mean CNR between LV myocardium with LVBP, RVBP, and lung in the MGE image series (Fig. 2c, d) were defined as optimum CNR images for composite image formation (Fig. 2e, f). The composite image (Fig. 2g, h) results from blending the registered optimum CNR images at equal weighting using the following equation:

3 $$C = \alpha + \beta + \gamma ,$$

where *C* corresponds to the composite image, and *α*, *β*, *γ* are the respective short-axis MGE images providing optimum CNR between LV myocardium and LVBP, RVBP, and lung.

### T2* quantification

Pixel-wise myocardial T2* was calculated for 4–6 segments per slice according to the American Heart Association (AHA) 16-segment model [2] using a monoexponential model with offset correction [12]:

4 $$y = Ke^{{{\hbox{${ - {\text{TE}}}$} \!\mathord{\left/ {\vphantom {{ - {\text{TE}}} {{\text{T}}2^{*} }}}\right.} \!\hbox{${{\text{T}}2^{*} }$}}}} + C ,$$

where *y*, *K*, TE, T2* and *C* represent signal intensity, a fitting constant, echo time, myocardium transverse relaxation time including the effect of field inhomogeneity, and a constant of offset correction (assumed to be zero, see below), respectively. For the segmental T2* analysis purposes, the AHA reference point for each slice was defined at the anterior-septal junction of LV and right ventricle [13] by the first author with more than 3 years cardiovascular imaging experience. In this study, the documentation of minimum segmental T2* value of short-axis images served to provide an indication of the presence of pathology where myocardial T2* ≤ 10 ms was considered as having severe myocardial iron, between 10 until 20 ms as having moderate myocardial iron, and >20 ms without iron loading [14, 15].

For noise determination, the evaluation of SNR of myocardium and an ROI of air background at every TE of all datasets was calculated using Eq. 1. Since in our data, even at the longest TE values, the SNR of the MGE images exceeded a value of 4, we can exclude that the T2* values calculated in monoexponential analysis were affected significantly by Rician noise [16, 17]. At SNR = 4 the correction schema proposed by Gudbjartsson et al. [18], would prescribe a small downward correction of MGE signal (−3.87 %). Therefore, Rician noise was not corrected for, and in our analyses Eq. 4 was reduced to [13]:

5 $$y = Ke^{{{\hbox{${ - {\text{TE}}}$} \!\mathord{\left/ {\vphantom {{ - {\text{TE}}} {{\text{T}}2^{*} }}}\right.} \!\hbox{${{\text{T}}2^{*} }$}}}} .$$

### Reproducibility evaluation of myocardial contouring

Four observers drew short-axis LV epicardial and endocardial contours by two procedures; method 1: the clinical standard method of drawing on one visually selected MGE image with good contrast (blind, independent selection by each observer) and method 2: our novel procedure of drawing contours on the contrast-optimized composite image. Two observers had more than 3 years cardiovascular imaging experience; the other two had less experience, nonetheless they had profound knowledge of short-axis myocardium anatomy.

Hand-tracing contours were drawn independently by the observers for each of the two methods using custom-written software described above with an interval of more than 2 weeks between methods. The LV endocardial region was drawn to encompass the entire blood pool with including papillary muscles and LV trabeculae. Optimum settings of window level and width were set by the first author for method 1 and defined automatically for method 2.

In method 2, selections of MGE images for maximum CNR between myocardium and its main surroundings were observer independent. Therefore in this study, the first author used the above mentioned semi-automated procedure to generate the composite images.

Lacking a gold standard, interobserver segmentation agreement of myocardial contours by the two methods was calculated using the dice similarity coefficient (DSC), which is a simple spatial overlap index that has been used for segmentation validation and shows stronger reflection of differences in location than differences in size [19, 20]. Between two sets of binary segmentation, a minimum DSC value of 0 indicates no spatial overlap referring to no contour agreement and a maximum value of 1 referring to a complete agreement [20]. DSC was defined as follows:

6 $${\text{DSC}} = (A,B) = \frac{2(A \cap B)}{(A + B)},$$

where *A*, *B* represent the contour regions and ∩, + represent the intersection and addition between regions, respectively [20].

### Statistical analysis

The ability of the contrast-optimized composite image in producing better CNR of myocardium and its main surroundings in one image was assessed by comparing its CNRs to the maximum CNRs between LV myocardium and LVBP, RVBP, and lung assessed on the MGE image series.

Myocardial contouring agreement between observers was assessed by the DSC and presented as medians ± median absolute deviations for all observers and observers with experience of more than 3 years and less in cardiovascular imaging. The Paired Wilcoxon test was used to compare the DSC agreement in myocardial contouring by the two methods. T2* value per segment was calculated by using Eq. 5 and presented as means ±1 SD. The interobserver reproducibility of the two methods was assessed using the Bland–Altman analysis [21] with ±1.96 SD as the limit of agreement (LoA). The two methods were compared with the same observer’s group as the DSC assessment.

On each short-axis slice, the minimum segment T2* value was identified to investigate iron deposition in patients with quantification by using method 2. Using this information, short-axis slices containing segments with T2* value <20 and >20 ms were grouped. In these slices' groups, myocardial contouring agreement and segmental T2* reproducibility of the two methods for all observers were also assessed using the Paired Wilcoxon test and Bland–Altman analysis.

The T2* quantification on the mid-ventricular septum and global heart regions was performed in this study on seven haematology patients with complete datasets of apical, mid-ventricular, and basal slices. The mid-ventricular septum was defined as the average of T2* values at mid-anterior septum and mid-inferior septum [9] and the global heart as the average of 16 segments previously described [2].

Variability between observers by using the two methods was presented as the Coefficient of variance (CoV), which is the standard deviation of the differences between observers in each method divided by their general mean and expressed as a percentage. Reliability of the methods as assessed by the observers was tested by using a two-way random intraclass correlation coefficient (ICC), evaluating absolute agreement Statistical analyses were performed using IBM SPSS Statistics software version 20 (IBM Corporation, Somers, NY, USA), *P* < 0.05 was considered statistically significant.

## Results

### T2* characteristics of patients

The minimum segmental T2* value per short-axis slice was used to assess (local) iron deposition in patients. Of the nine haematology patients, seven patients with the complete three slices had the minimum T2* in the range of 10.48–26.33 ms at apical, 8.20–27.97 ms at mid-ventricular, and 7.05–25.65 ms at basal, while two others measured at the mid-ventricular had minimum segmental T2* values of 12.72 and 17.78 ms. For the twelve suspected cardiomyopathy patients, the range of minimum segmental T2* at mid-ventricular was 12.42–36.75 ms (*n* = 12) and at basal 26.95 ms (*n* = 1).

In all 36 datasets of 21 patients, the minimum segmental T2* values <20 ms were encountered in 14 datasets of seven haematology and three suspected cardiomyopathy patients (three apical, seven mid, and four basal) in which on one haematology patient the minimum segmental T2* values <10 ms encountered in apical, mid-ventricular, and basal slices. The minimum segmental T2* values <20 ms was commonly encountered at anterior and inferior segments in apical; at anterior, inferolateral and anterolateral slices in mid-ventricular; and at anterior, anteroseptal and inferolateral in basal slices.

### CNR evaluation

The contrast-optimized composite images were produced, as shown in Fig. 2, by combining those images providing maximum CNR between LV myocardium, LVBP, RVBP, and lung, generally two or three different images from the MGE series (56.71 and 41.90 % respectively). In this study, maximum CNR between LV myocardium and LVBP commonly occurred at later TE: 15.97 ms (23.40 %) and 18.20 ms (50.90 %). The pattern between LV myocardium and RVBP was similar at 15.97 ms (14.40 %) and 18.20 ms (29.90 %), while maximum CNR between LV myocardium and lung commonly happened at early TE: 2.59 ms (30.10 %) and 4.82 ms (30.80 %). Since the custom-written software only needs 1 s to generate the composite image, the total time to produce a semiautomatic composite image depends on the time needed by the observer to draw the LV epicardium, LVBP, RVBP, and lung adjacent to the myocardium (about 15 s).

In method 2, the semi-automatic analysis yielded reproducible improvements of CNR on the composite image as compared with the original MGE images. Beyond the maximum value measured at any MGE image, the composite image increased contrast by an average of 12.08 % between myocardium and LVBP (95 % CI 9.22–14.94 %; ICC of 0.89); 9.74 % between myocardium and RVBP (95 % CI 6.26–13.22 %; ICC of 0.83); and 30.33 % between myocardium and lung (95 % CI 28.21–32.45 %; ICC of 0.72). On average, between myocardium and its main surroundings, the contrast improvement was 17.38 % (95 % CI 15.64–19.13 %; ICC of 0.87) above maximum CNR at any TE.

### Interobserver reproducibility of myocardial contouring

The total of 36 short-axis slices yields a set of 36 DSCs of myocardial contours of short-axis images for each method, and the combination of four observers thus adds up to sets of (36 × 6 = 216) DSCs contours. Between all observers, interobserver reproducibility of myocardial contouring improved when using contrast-optimized composite images (method 2) compared to visually selected images (method 1) with DSC of 0.844 ± 0.032 vs. 0.811 ± 0.045, *P* < 0.001. Between observers with more than 3 years cardiovascular imaging experience, the improvements also were significant (0.871 ± 0.017 vs. 0.829 ± 0.033, *P* < 0.003), as were those between observers with less experience in cardiovascular imaging (0.844 ± 0.034 vs. 0.783 ± 0.056, *P* < 0.003). As expected, the respective dice coefficients were slightly lower for the less experienced observers. On short-axis slices having segments with minimum T2* < 20 and >20 ms, interobserver reproducibility improved similarly when using method 2 compared to method 1 (0.842 vs. 0.822, *P* < 0.001 and 0.847 vs. 0.804, *P* < 0.001, respectively). When myocardial contouring was done by method 1, the most frequently selected MGE images were those of TE 9.28 ms (29.9 %), 7.05 ms (24.3 %), and 4.82 ms (22.9 %). In method 1, reproducibility of contours tended to be less between observers selecting a different image for ROI drawing (*P* < 0.001, data not shown).

### Interobserver reproducibility of segmental myocardial T2*

**Fig. 3:**
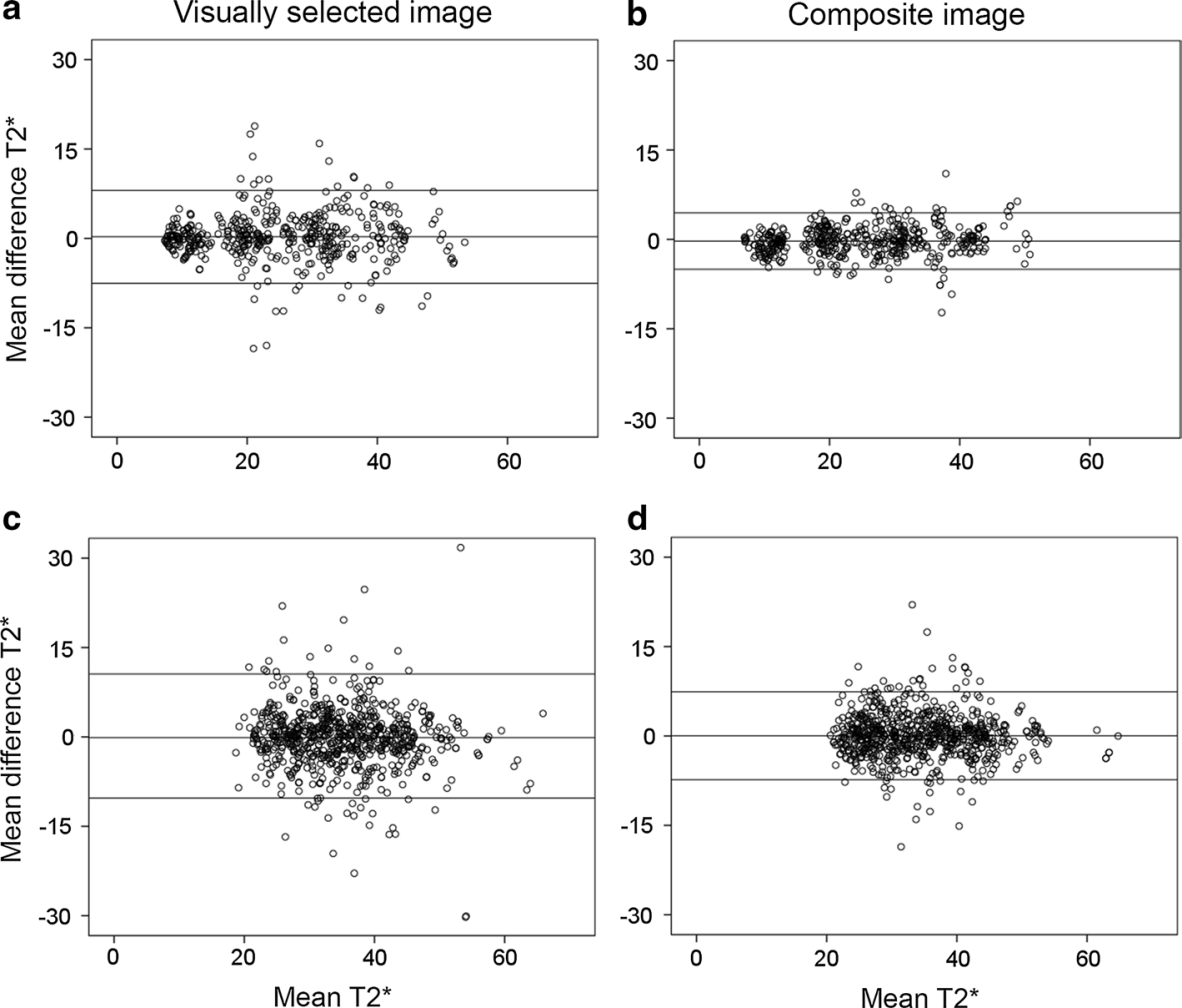
Bland–Altman plots between observers assessing myocardial T2* on short-axis slices. **a**, **b**: Assessed on **a** visually selected multi gradient echo image (MGE) (method 1) and **b** contrast-optimized composite image (method 2) in the presence of a segment with minimum T2* < 20 ms (468 segments). **c**, **d**: Assessed on **c** visually selected MGE image and **d** contrast-optimized composite image (method 2) in the absence of segments with minimum T2* < 20 ms (744 segments)

A total of 36 short-axis slices yields two sets of 202 segments of myocardial T2* values between two observers (7 × 4 apical + 29 × 6 mid/basal) and the combination of four observers, thus, adding up to two sets of (202 × 6 = 1212) segments of myocardial T2* values for each method. Standard deviations of all T2* values listed in Table 1, are consistently smaller for method 2 than for method 1. Table 1 shows the results of Bland–Altman analysis for myocardial T2* per segment applied on all 36 slices of 21 patients by both methods. T2* quantification per segment between all observers using method 2 shows a better interobserver reproducibility with lower LoA and CoV compared to method 1 (LoA; CoV; ICC of ±6.49; 10.81 %; 0.987 vs. ±9.32; 15.25 %; 0.975). The same trend is seen between observers with more than 3 years (LoA; CoV; ICC of ±5.40; 8.63 %; 0.966 vs. ±6.53; 10.64 %; 0.954) and less experience in cardiovascular imaging (LoA; CoV; ICC of ±5.41; 9.19 %; 0.965 vs. ±10.03; 16.79 %; 0.895). For the T2* quantification per segment between all observers on 14 short-axis slices with minimum segment T2* < 20 ms (Table 1; Fig. 3a, b), method 2 reveals lower CoV than method 1 (LoA; CoV; ICC of ±4.73; 9.79 %; 0.994 vs. ±7.80; 15.91 %; 0.984) with the same trend on 22 short-axis slices with minimum segment T2* > 20 ms (Table 1; Fig. 3c, d) (LoA; CoV; ICC of ±7.38; 10.94 %; 0.973 vs. ±10.16; 14.77 %; 0.948).

**Table 1:** Bland–Altman analysis of T2* value per segment between observers which was assessed by using visually selected (M1) and contrast-optimized composite (M2) images

Comparison of T2* value per segment on short-axis slices	ns	Mean ± SD (ms)		Mean difference ± SD (ms)		LoA		CoV (%)		ICC	
		M1	M2	M1	M2	M1	M2	M1	M2	M1	M2
All slices examined by all observers	1212	31.20 ± 10.72	30.65 ± 10.40	0.03 ± 4.76	−0.09 ± 3.31	±9.32	±6.49	15.25	10.81	0.975	0.987
All slices examined by observers >3 years experience	202	31.93 ± 10.86	31.30 ± 10.52	−0.18 ± 3.33	−0.31 ± 2.76	±6.53	±5.40	10.64	8.63	0.954	0.966
All slices examined by observers <3 years experience	202	30.46 ± 10.66	30.00 ± 10.37	0.75 ± 5.12	0.38 ± 2.76	±10.03	±5.41	16.79	9.19	0.895	0.965
Slices with minimum segment T2* < 20 ms	468	25.00 ± 11.29	24.67 ± 10.83	0.27 ± 3.98	−0.29 ± 2.41	±7.80	±4.73	15.91	9.79	0.984	0.994
Slices with minimum segment T2* > 20 ms	744	35.10 ± 8.24	34.42 ± 8.12	−0.11 ± 5.18	0.04 ± 3.77	±10.16	±7.38	14.77	10.94	0.948	0.973

*ns* number of segment, *SD* standard deviation, *ms* millisecond, *LoA* limit of agreement, *CoV* coefficient of variation, *ICC* intraclass correlation

In Table 2, the global heart and mid-ventricular septum T2* analysis was done on seven haematology patients who had complete basal, mid-ventricular, and apical slices by using the two methods. Method 2 has improved interobserver reproducibility for all observers compared to method 1 in assessing T2* on the global heart region (CoV; ICC of 12.29 %; 0.987 vs. 16.88 %; 0.976) as well as mid-ventricular septum region (CoV; ICC of 5.76 %; 0.998 vs. 8.16 %; 0.995). These improvements were also seen in observers with either high or low experience in cardiovascular imaging.

**Table 2:** Coefficient of variation (CoV) and intraclass correlation (ICC) for global myocardium and mid-ventricular septum T2* values assessed by using visually selected (M1) and contrast-optimized composite (M2) images

	Global heart T2*				Mid-ventricular septum T2*			
	M1		M2		M1		M2	
	CoV (%)	ICC	CoV (%)	ICC	CoV (%)	ICC	CoV (%)	ICC
Examined by all observers	16.88	0.976	12.29	0.987	8.16	0.995	5.76	0.998
Examined by >3 years experience observers	11.47	0.956	8.99	0.971	7.77	0.982	5.52	0.987
Examined by <3 years experience observers	19.41	0.886	10.09	0.967	9.80	0.973	6.18	0.990

T2* analysis of all 16 myocardial segments as assessed by all observers on all 36 short-axis slices of the 21 patients is shown in Table 3. In this analysis, higher interobserver reproducibility of T2* per segment is shown by method 2 with lower CoV and higher ICC compared to method 1 in most of the 16 segments. When the T2* analysis of the mid-ventricular slice is extended from 7 (Table 2) to 21 slices, higher interobserver reproducibility by method 2 is retained in the mid-ventricular anteroseptal (CoV; ICC of 6.19 %; 0.995 vs. 7.87 %; 0.992) and inferoseptal (CoV; ICC of 5.66 %; 0.994 vs. 8.57 %; 0.986) segments corresponding with the mid-ventricular septum.

**Table 3:** Coefficient of variance (CoV), and intraclass correlation (ICC) for segmental T2* assessed in 16 myocardial segments using visually selected (M1) and contrast-optimized composite (M2) images

Myocardial segments		M1		M2	
		CoV (%)	ICC	CoV (%)	ICC
Basal					
1	Anterior	16.32	0.969	14.99	0.965
2	Anteroseptal	9.40	0.992	9.48	0.991
3	Inferoseptal	23.62	0.960	6.52	0.996
4	Inferior	19.37	0.957	9.89	0.989
5	Inferolateral	15.18	0.967	10.70	0.982
6	Anterolateral	16.02	0.966	11.30	0.984
Mid-ventricular					
7	Anterior	11.20	0.985	10.61	0.989
8	Anteroseptal	7.87	0.992	6.19	0.995
9	Inferoseptal	8.57	0.986	5.66	0.994
10	Inferior	17.24	0.965	11.86	0.983
11	Inferolateral	22.42	0.933	15.10	0.972
12	Anterolateral	17.32	0.965	14.98	0.970
Apical					
13	Anterior	21.25	0.912	14.22	0.950
14	Septal	8.94	0.992	7.01	0.995
15	Inferior	19.26	0.975	9.94	0.993
16	Lateral	12.08	0.987	12.89	0.986

## Discussion

This study shows on average 17 % CNR improvement using the proposed contrast-optimized composite image (method 2) over any maximum contrast provided by the single image technique (method 1), providing reproducible T2* values in short-axis slices of the mid-ventricular septum as well as global heart regions. These findings may be beneficial for the use of T2* imaging in the diagnosis of thalassemia and suspected cardiomyopathy patients with borderline myocardial T2* distribution. Iron overload may be heterogeneous with septal T2* values not necessarily representative for the whole left ventricular myocardium [2].

A recent study by House et al. [22] on the post mortem heart reveals segmental variation of iron loading other than septal, especially in the lateral wall, with different distribution on epicardial and endocardial regions confirming previous findings [23, 24]. Therefore, in this study, segmental T2* was assessed for all myocardial segments rather than a c-shaped area with two arbitrary cut-offs, which, although frequently used in previous studies [1], may be erroneous. Note that beside iron content, T2* variations between myocardial segments may also reflect the different orientations of the myocardial capillaries to the external magnetic field, resulting in locally varying susceptibility effects and consequently varying degrees of dephasing [25]. However, findings by Meloni et al. [3, 9], with use of artifact correction maps, indicate that neither additive susceptibility artifacts nor blood oxygenation significantly affects the global heart T2*.

Evaluation of the T2* quantification at mid-ventricular septum and global heart regions (Table 2) shows that method 2 improves the interobserver reproducibility (lower CoV and higher ICC) compared to method 1 showing the superiority of contrast-optimized composite images from a single TE image for clinical T2* quantification. Looking at the 16 myocardial segments (Table 3), T2* quantification by using method 2 also shows lower CoV and higher ICC compared to method 1 in most segments.

Comparing the results using method 2 to other research groups for global heart T2* analysis (Table 2), similar interobserver reproducibility was noted by one research group using a single TE image on its myocardial contouring [26]. While at mid-ventricular septum T2* analysis, higher interobserver reproducibility was achieved by using method 2 compared to other research groups, which used a single TE image [5, 26]. If the comparison is broken down into 16 segments T2* rather than global heart T2* (Table 3), the interobserver reproducibility by using method 2 has shown a higher interobserver reproducibility, compared to the selection of one MGE image by another research group [26]. Moreover, the selection of a good contrast (higher TE) image by method 1 provides, in overall, a better interobserver reproducibility compared to others using the first or second (short TE) image [3, 26]. This may reflect the fact that in this study, even at higher TE’s, the MGE images still had SNR > 4.

It was shown in this study that maximum contrast between myocardium and its surrounding borders is not likely to occur on a single MGE image. The observer’s preference in selecting an MGE image having a good contrast also reveals a range of TE values between 4.82 and 9.28 ms. The observer dependent difference in image selection did significantly contribute to the reduction of observers agreement on myocardial contouring (DSC value, *P* < 0.001) leading to higher variability (CoV) and lower reliability (ICC) in T2* quantification in this study (Method 1) (data not shown).

In this study, myocardial contouring was performed by the normal standard method (method 1) and by our new method (method 2). In general, both methods showed good interobserver reproducibility with good overlap of myocardial contour drawing. However, even better reproducibility was obtained by method 2(*P* < 0.001) especially for experienced observers getting DSC above 0.85. This demonstrates the consistency of myocardial information acquired by the method [19]. This result is in line with the reductions of T2*’s standard deviation, limits of agreement, and coefficients of variance also with the higher intra class correlation evaluates between observers using method 2 compared to method 1 (Table 1).

This study documented the minimum segment T2* values in order to provide an indication of the presence of pathology, such as an increased risk of heart failure at T2* < 20 ms [14]. In slices without any segment meeting T2* < 20 ms, i.e., without an indication of iron accumulation in the left ventricular myocardium, the DSC values by the two methods showed good contour overlaps between observers but higher DSC values were reached by using method 2 (*P* < 0.001). Importantly, in the remaining slices with segments of T2* < 20 ms indicating iron deposition as a result of pathology, the respective values were similar (*P* < 0.001). These results thus indicate that our newly proposed composite image method not only gives improvement in “normals” but also improves the reproducibility of myocardial contours in the presence of iron deposition as a result of focal pathology.

In this study we thus demonstrated that the use of a contrast-optimized composite image derived from the MGE image series, as a reference image for drawing myocardial contours, leads to higher segmental T2* value reproducibility compared to the use of one visually selected MGE image. The composite image is able to optimize the contrast between the myocardium and its surroundings (mainly LVBP, RVBP and lung tissue) on one image with even higher CNR (17 % in this study) than any individual maximum contrast between the myocardium and its surroundings, which are unlikely to be reached at one TE value. An equal weighting factor to generate contrast-optimized composite image (Eq. 3) is used in this study to maintain equal contrast contribution between the myocardium and its main surroundings provided by the MGE images. Seeing that maximum CNR between LV myocardium, LVBP, and RVBP (α and β in Eq. 3) happen with the same pattern on MGE images at the later TE while between LV myocardium and lung (γ in Eq. 3) happens mostly at the early TE, a combination with different weighting factors for α, β and γ can be a future direction to optimized contrast on the composite image. The short time needed for generating the composite image (on average 15 s) turns our proposed method into a good alternative for providing reference images for myocardial contour drawings in the clinical practice. The Bland–Altman limit of agreement (Table 1) show that the use of a contrast-optimized composite image is able to produce more accurate and reliable segmental T2* values obtained by different observers with lower CoV and higher ICC. Moreover, the use of the proposed contrast-optimized composite image also improves the ability of less experienced observers in assessing reproducible segmental T2*.

Our novel approach has the limitation that it was done on a small number of patient data for methodological purpose. A validation on a larger patient data base is encouraged to confirm the advantages of our new analysis method in clinical practice. The approach still requires manual drawing of the myocardial and its surrounding contours for composite image formation, which gives an additional task in the clinical routine workflow. Moreover, the ROIs of lung and air background must be carefully positioned to avoid any influence of blood vessels and ghosting artefacts. However, we expect that in the near future standardizing ROI drawing in an automatic manner will minimize this limitation. The MGE MRI protocol of this study did not include parallel imaging acquisition. In the latter case (as also to some extent with our use of phased array coils) uneven k-space sampling would make noise estimates tricky. However, in parallel imaging MGE studies the use of relative CNR’s rather than absolute CNR’s (Eq. 2) would be a valid alternative still allowing for wide implementation of our newly proposed method. Elsewhere, an automatic segmentation method for myocardial contouring showed highly improved reproducibility for T2* quantification for images showing high contrast between the myocardium and its surroundings as in black blood T2* acquisition [27]. An evaluation of the use of contrast-optimized composite images in black blood T2* acquisition would be needed to investigate the advantage of our method in producing more reproducible segmentation on the black blood image (beyond the scope of the current study). The development and implementation of automatic myocardial segmentation rather than manual ROI drawing on bright blood T2* MGE images, using the composite image method, are the topic of ongoing investigations at our institution.

## Conclusions

In conclusion, the proposed contrast-optimized composite method has proven to be effective by providing highly reproducible myocardial contours yielding more accurate T2* measurements not only in the presence of myocardial iron accumulation but also in global heart T2* and mid-ventricular septum T2* analysis. This new method profits from a standardized generation of a composite bright blood T2* MGE image, with on average 17 % improved contrast to noise ratio between LV myocardium and the surrounding tissues. This improves reproducibility of myocardial contour determination, leading to increased consistency in the calculated T2* values enhancing the diagnostic impact of this measure of iron overload.
